# A parameterized model for tower crane energy consumption was developed based on theoretical formulation and field data

**DOI:** 10.1038/s41598-025-94875-5

**Published:** 2025-03-26

**Authors:** Fan Zhang, Chunli Zhang, Yan Fu, Jun Liu, Jiarui Bu, Peng Duan, Si Chen

**Affiliations:** 1 School of Management Science and Real Estate, Chongqing University, Chongqing, 400045 China; 2 Center for Construction Economics and Management, Chongqing University, Chongqing, 400045 China; 3 Institute of Urban-rural Construction and Development, Chongqing University, Chongqing, 400045 China; 4 Chongqing Jianzhu College, Chongqing, 400072 China; 5 China Construction Second Engineering Bureau Ltd., Beijing, 100160 China

**Keywords:** Energy consumption, Tower crane, Partial least squares regression, Calculation accuracy, Civil engineering, Statistics

## Abstract

As tower cranes (TC) getting more use in the construction process, a reliable TC energy consumption calculation model is increasingly required for construction management. This paper proposed a semi-empirical model, which is based on the division of TC work cycle. For fitting the coefficients, Partial Least Squares Regression (PLSR) was adopted. To simplify the model, variables with weak regression significance to energy consumption were deleted in turn. The best suitable version achieves a Mean Absolute Percentage Error of 25.55%, a Root Mean Square Error (RMSE) of 1036.19 kJ, and a Coefficient of Determination (R^2^) of 0.83, with just one independent variable. A comparative analysis showed the proposed model had the highest accuracy and fitting degree among all the models for TC energy consumption calculation. Through physical transformation of the proposed model, several key engineering parameters (i.e., load mass, number of work cycles, and hoisting height) affecting TC energy consumption were extracted. The innovation of this empirical study lies in confirming the feasibility of the stage-based calculation model and the small sample fitting strategy, providing new ideas of constructing and optimizing energy consumption models for other construction machinery. At the same time, the proposed model lays a foundation for research related to TC energy consumption to be more reliable.

**Supplementary Information:**

The online version contains supplementary material available at 10.1038/s41598-025-94875-5.

## Introduction

The construction industry has a significant impact on the environment and ecology^1^. Approximately one third of the global energy consumption comes from the construction industry, and the proportion is on the rise^2^. Stimulated by environmental regulations and national demands, government departments in many countries have to strengthen the control of energy consumption in the construction industry^3,4^. Under such circumstances, energy consumption has become an important factor affecting the development of construction enterprises^5^. Since construction machinery is the main source of energy consumption in the construction phase of the entire building life cycle^6–8^, it’s very important to find methods that can accurately calculate or predict the energy consumption of construction machinery for a better management work in the construction phase.

Recently, research on the energy consumption of important construction machinery is being continuously improved^6,9–11^. However, there is a significant lack of research on the energy consumption of tower cranes (TC). On the one hand, due to the promotion of prefabricated and modular buildings, the application amount of tower cranes on construction sites has generally shown an increasing trend in recent years^12–14^. On the other hand, energy consumption is an important decision making factor for issues such as the site layout and hoisting strategy of TC^15,16^. Therefore, it is necessary to conduct research on TC energy consumption.

Proposing a semi-empirical energy consumption calculation model for TC based on the external motion stages is the most critical innovation of this study. Because of omitting the complexity of the internal mechanisms, and using the Partial Least Squares Regression (PLSR) method to calculate the empirical coefficients, the model is easy to use in practical scenarios. In the proposed semi-empirical model, the TC’s operation process is divided into 6 stages, which significantly improves the accuracy of energy consumption calculation. In addition, this study is based on measured data, which is different from most of TC’s research.

The remainder of this paper is organized as follows: Sect. "Literature review" provides a literature review mainly about construction machinery energy consumption model types and construction methods. The methods used in the study is described in Sect. "Materials and methods", mainly expounding the division principle of TC’s operation process, the data set and the regression method. Section "Results" is about the fitting results and some basic elaboration. Section "Discussion" further discusses and analyzes the tower crane energy consumption calculation model. In Sect. "Theoretical and practical implications", the significance of the study is summarized. Finally, Sect. "Conclusions" concludes the main findings, emphasizes the potential limitations, and provides suggestions for subsequent research. This study aims to provide an easy to use and highly accurate tower crane energy consumption calculation method to support more effective construction management and research.

## Literature review

The construction machinery energy consumption is an important object for calculating the energy consumption and carbon emissions of whole building life cycle^17^, and is also one of the optimization objects of engineering project management^18–20^. The application performance of its calculation model significantly affects the quality and effect of research and work of engineering energy consumption management. Therefore, how to improve the accuracy and practicality of construction machinery energy consumption calculation models has become a crucial topic in many studies^21,22^.

“Power × Time = Energy” has been one of the basic methods for calculating energy consumption. Usually, this formula is expressed as “Mechanical Rated Power × Mechanical Operation Time = Energy Consumption”^23–26^. It can be called Empirical Model since the power data of the machinery usually comes from equipment suppliers or quota documents^25,27^. In order to reduce the calculation error, correction factors are applied to adjust the calculation results^24^. Even though Empirical Model is very simple in form, its calculation results may not be reliable^28^. Recording the power fluctuations during the entire operation of construction machinery and using integration to calculate energy consumption is a more reliable method^29,30^, but it’s only suitable for controlled laboratory environments, there is always limitation on data collection in complex construction environments^27^. Currently, most researchers are still relying on the first, error-prone but simple method for calculating energy consumption. In some studies on other machinery, the operational process has been divided into several stages, and the total energy consumption has been accumulated from each stage, with different power functions for each phase^31,32^. This approach is worth attempting for construction machinery, though its applicability and accuracy haven’t been discussed yet.

Another method for calculating energy consumption depends on the relationship between engineering parameters and energy consumption. Load is generally considered a key factor affecting the energy consumption of construction machinery, so in some studies, load mass has been treated as an important parameter for energy consumption calculation^33,34^. Recent research suggests that, aside from load, other engineering parameters can also provide reliable predictions for the energy consumption of specific machinery^9,35^.

Although energy consumption issues have been widely involved in the field of construction machinery, there is still a limitation in the types of energy consumption calculation models being applied, and there seems to be a lack of analysis on the accuracy of different models. This may make it difficult to select the appropriate model for practical applications. Furthermore, although some studies have begun to construct functional relationships between construction machinery energy consumption and engineering parameters, there has been little clarity on how to select key parameters from a multitude of factors. Developing a method for identifying critical parameters could greatly promote future research on construction machinery energy consumption.

There are numerous methods for determining coefficients in energy consumption models, which can generally be categorized as white-box, gray-box, and black-box methods. White-box methods calculate coefficients using detailed physical information and logic, while black-box methods (such as Artificial Neural Network (ANN), Support Vector Machine (SVM), and regression statistics) fit coefficients based on historical data. Gray-box methods combine the features of the above two approaches^36^. Studies suggest that models based on historical data fitting have significant advantages in terms of cost, accuracy, and computational speed. ANN, SVM, and other methods are especially effective in handling cases where parameter relationships are unclear^19,34,37–40^, but they require large sample sizes and their computational efficiency is generally moderate^41,42^. Partial Least Squares Regression (PLSR) is an outstanding method in regression statistics, particularly effective in handling multicollinearity and small sample issues. At present, PLSR has been rarely applied in research on machinery energy consumption prediction, but existing studies have shown its good model fitting ability^28^. Moreover, in recent years, energy consumption models built using field data have gained attention in the study of construction machinery^43,44^. While collecting field data is challenging, it is more representative of the machinery’s actual operational conditions than laboratory or simulation data^45,46^.

Although numerous studies have confirmed the powerful capabilities of ANN, SVM, and similar methods in fitting coefficients for energy consumption models, there is no evidence indicating significant flaws in regression-based methods, such as PLSR, when fitting coefficients. Many studies may have overlooked the simplicity advantages of statistical regression methods in this area. Moreover, research on TC in particular currently lacks sufficient measured data support, and analyzing measured data could improve the applicability of research findings in real-world scenarios.

In summary, the core task of this research is to provide a reliable and practical energy consumption calculation model for future research in the field of TC. This study is based on two key assumptions: (a) Each work stage of TC has a relatively stable nominal power. (b) PLSR method is applicable to building energy consumption model coefficients with small samples for tower cranes. Given the limitations of existing research in model construction and fitting methods, this study aims to develop a TC energy consumption model that considers power differences during various work stages. It will use PLSR method to fit coefficients and validate the correctness of the assumptions and the accuracy of the model using measured data. Additionally, this study proposes a new approach to explore the relationship between construction machinery energy consumption and other engineering parameters, which accomplished by transforming the expressions of time and power in the semi-empirical model. This research confirms the feasibility of applying both the stage-based energy consumption model and PLSR method to energy consumption management research in construction machinery, and provides a practical approach for identifying key engineering parameters that affect the energy consumption of other machinery in future studies.

## Materials and methods

**Fig. 1:**
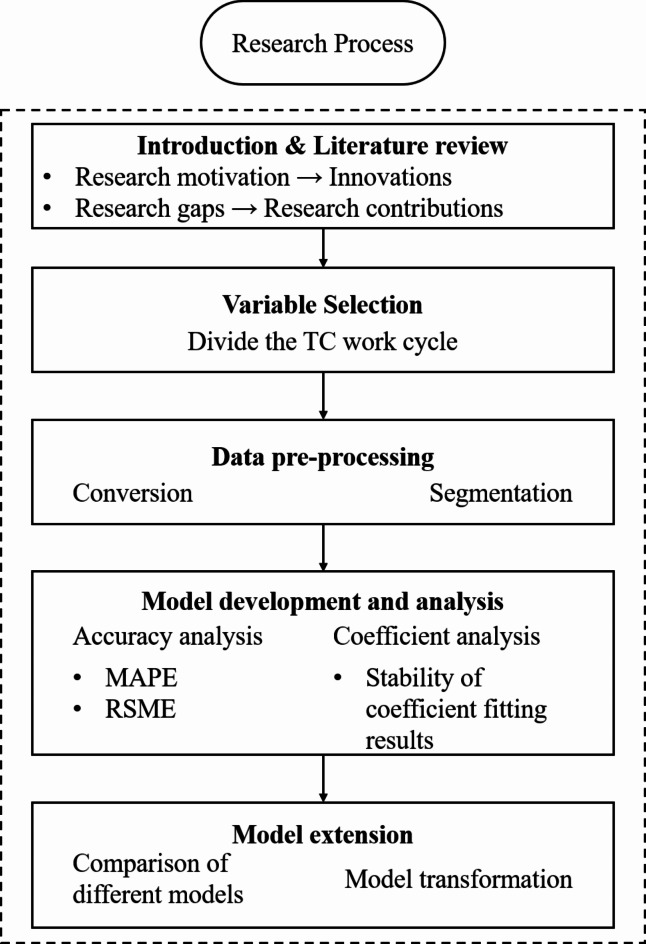
Research process of TC energy consumption model.

After literature review, the research process can be divided into four steps. The first step involves proposing a reasonable classification scheme for TC work stages. The second step includes the description of the measured data for model coefficient fitting. The third step is model fitting, evaluation, and analysis of the characteristics in the model. The last step focuses on improving model practicality, and uncovering deeper patterns and characteristics. Figure 1 provides an overview of the research process. This section mainly discusses the methods for the first and second steps, while latter steps correspond to Sect. "Results" and Sect. "Discussion".

### Variable selection

During operation, the tower crane continuously repeats the process of lifting items from the supply point to the demand point, and then returning empty from the demand point to the supply point. This entire process is referred to as a TC work cycle in this study. The work of TC consists of multiple work cycles, making the work cycle a suitable object for calculating TC energy consumption.

How to divide TC work stages is a key issue in this research, as it directly impacts the accuracy of the energy consumption calculation model. So far, there is no universally accepted classification scheme^47^. The division proposed in this study is based on two main reasons: (a) whether TC is loaded, which could affect the machinery’s energy consumption, and (b) the hoisting, slewing, and trolleying mechanisms are the main contributors to TC energy consumption, and there are significant differences in power usage across these mechanisms. Based on these factors, the stage division scheme shown in Table 1 is proposed.

**Table 1:** Stage division scheme for TC work cycle.

Stage name	Abbreviation	Whether loaded or not	Main energy consumption contribution mechanisms
Loading stage	LO	Unloaded→loaded	–
Hoisting stage	HS	Loaded	Hoisting mechanism
Horizontal transportation stage	HT	Loaded	Slewing mechanism, Trolleying mechanism
Descending stage	DE	Loaded	–
Unloading stage	UL	Loaded→unloaded	–
Return stage	RT	Unloaded	Hoisting mechanism, Slewing mechanism, trolleying mechanism

This study assumes that there are significant power differences in each of the stages shown in Table 1. The operating time for each stage is considered a variable in this study’s model. The energy consumption calculation model for a single work cycle is as follows:

1 $$E={\hat {P}_{{\text{LO}}}} \times {t_{{\text{LO}}}}+{\hat {P}_{{\text{HS}}}} \times {t_{{\text{HS}}}}+{\hat {P}_{{\text{HT}}}} \times {t_{{\text{HT}}}}+{\hat {P}_{{\text{DE}}}} \times {t_{{\text{DE}}}}+{\hat {P}_{{\text{UL}}}} \times {t_{{\text{UL}}}}+{\hat {P}_{{\text{RT}}}} \times {t_{{\text{RT}}}}$$

Where *E* (kJ) and  ${\hat {P}_{\text{X}}}$ (kW) in Function (1) represent the total energy consumption calculation of a work cycle and the empirical power coefficient of the X stage, respectively.

**Fig. 2:**
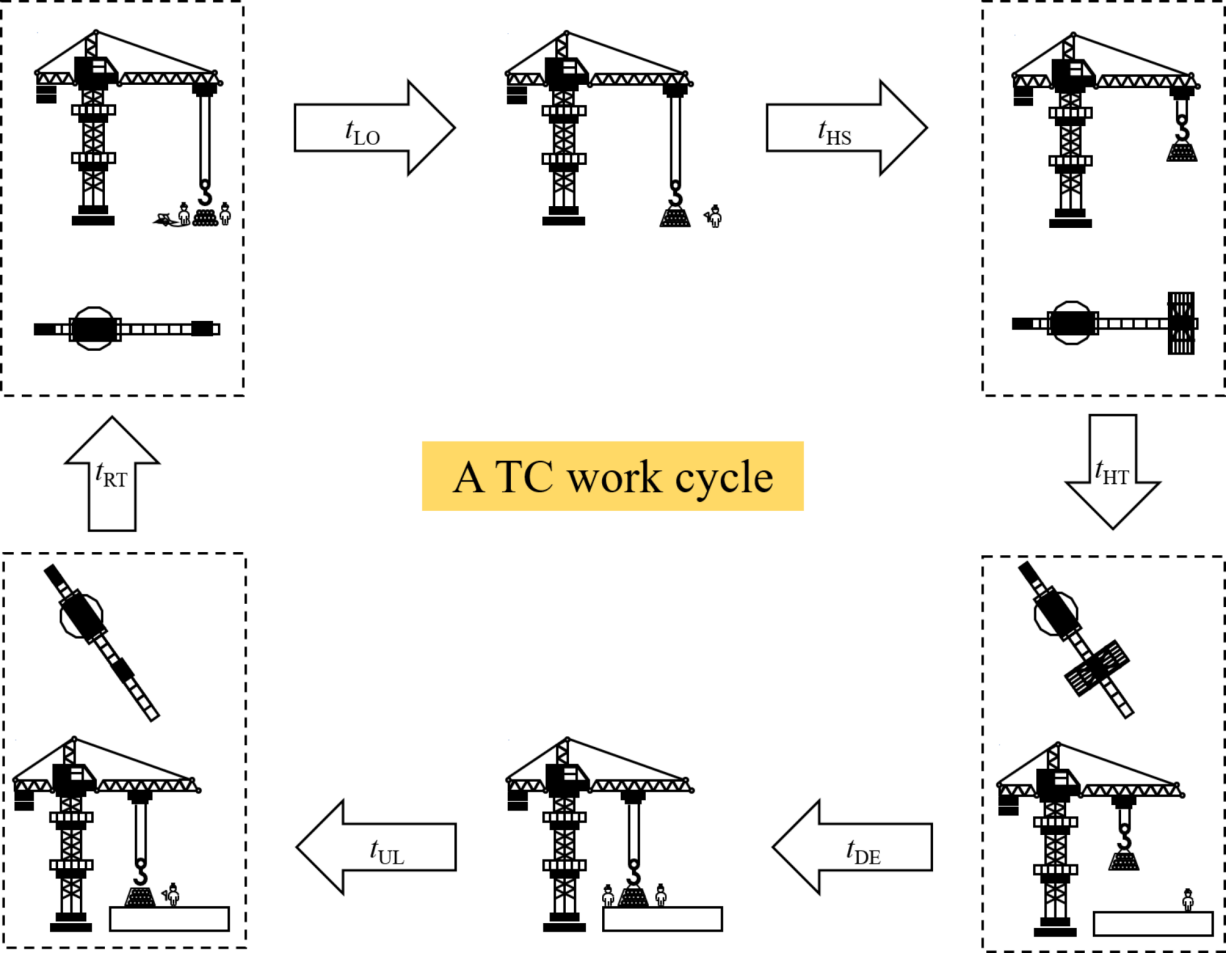
Division of a TC work cycle.

The time data for each stage is determined by TC external motion state, with the following rules (Fig. 2):

*t*_LO_ – Loading Time: Starts when workers attach the load, ending when the operator receives the signal to begin hoisting.

*t*_HS_ – Hoisting Time: Begins when the hoisting lever is pulled, and ends when the hook stops moving vertically.

*t*_HT_ – Horizontal Transportation Time: Starts when the hook’s horizontal position changes, ending when it stops moving horizontally.

*t*_DE_ – Descending Time: Starts when the hook begins to descend, ending when it stops moving vertically.

*t*_UL_ – Unloading Time: Begins when the hook stops moving, ending when unloading is complete and the crane moves to the next point.

*t*_RT_– Return Time: Starts when the hook’s position changes to return to the loading point, ending when the hook stops moving.

**Fig. 3:**
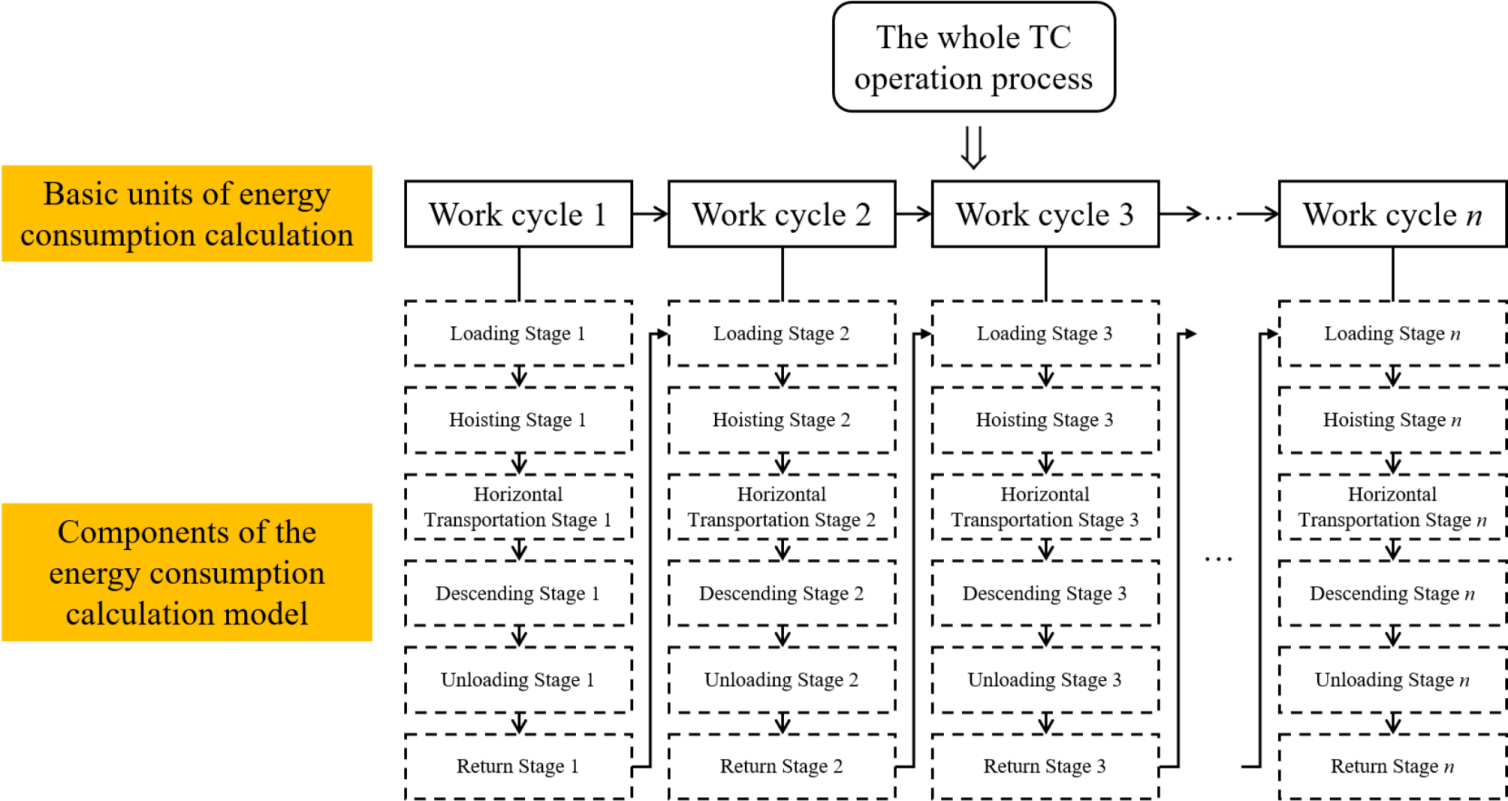
Energy consumption calculation’s architecture.

Figure 3 shows the architecture of TC energy consumption calculation in this study.

### Data preparation

**Fig. 4:**
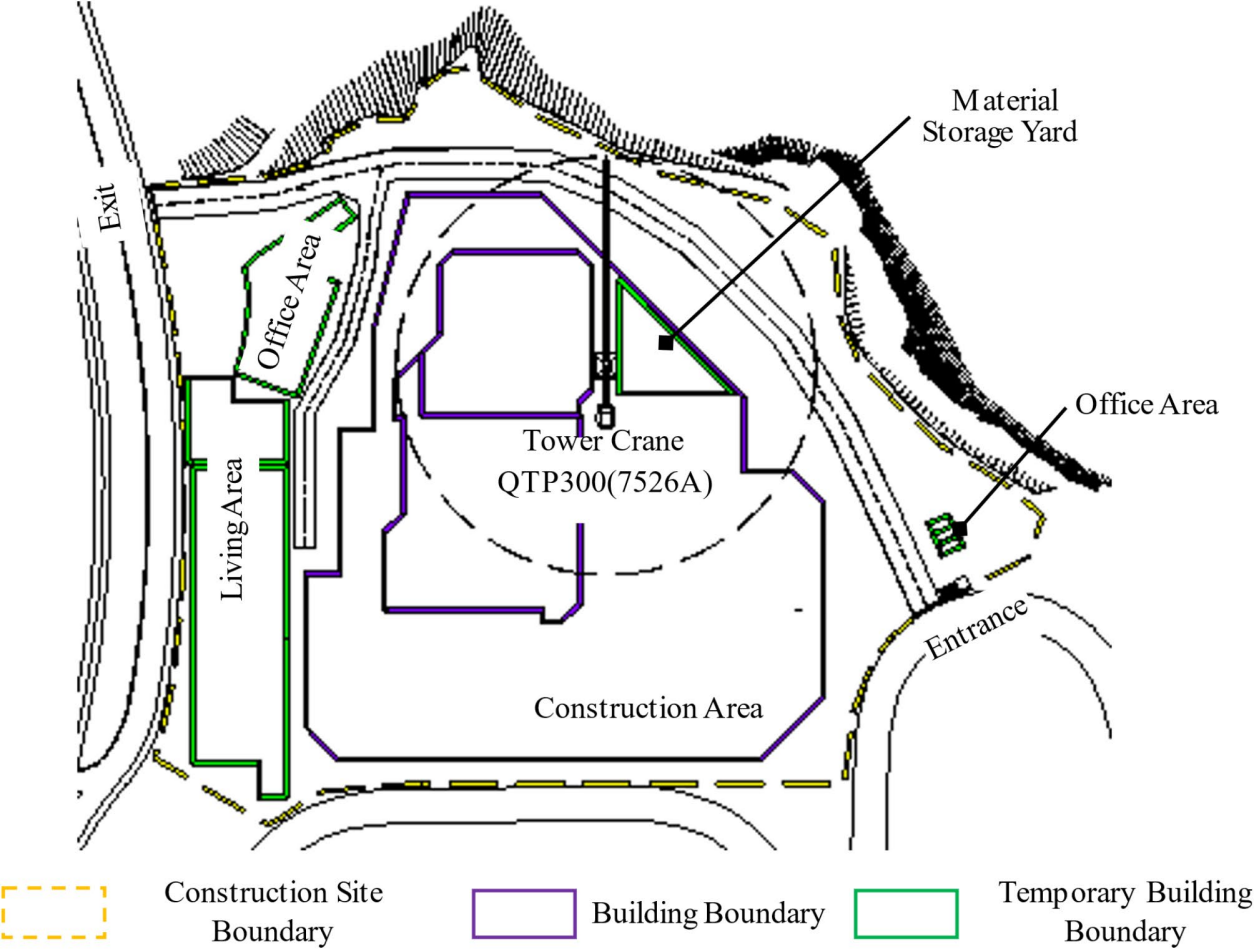
Simplified construction site layout.

**Fig. 5:**
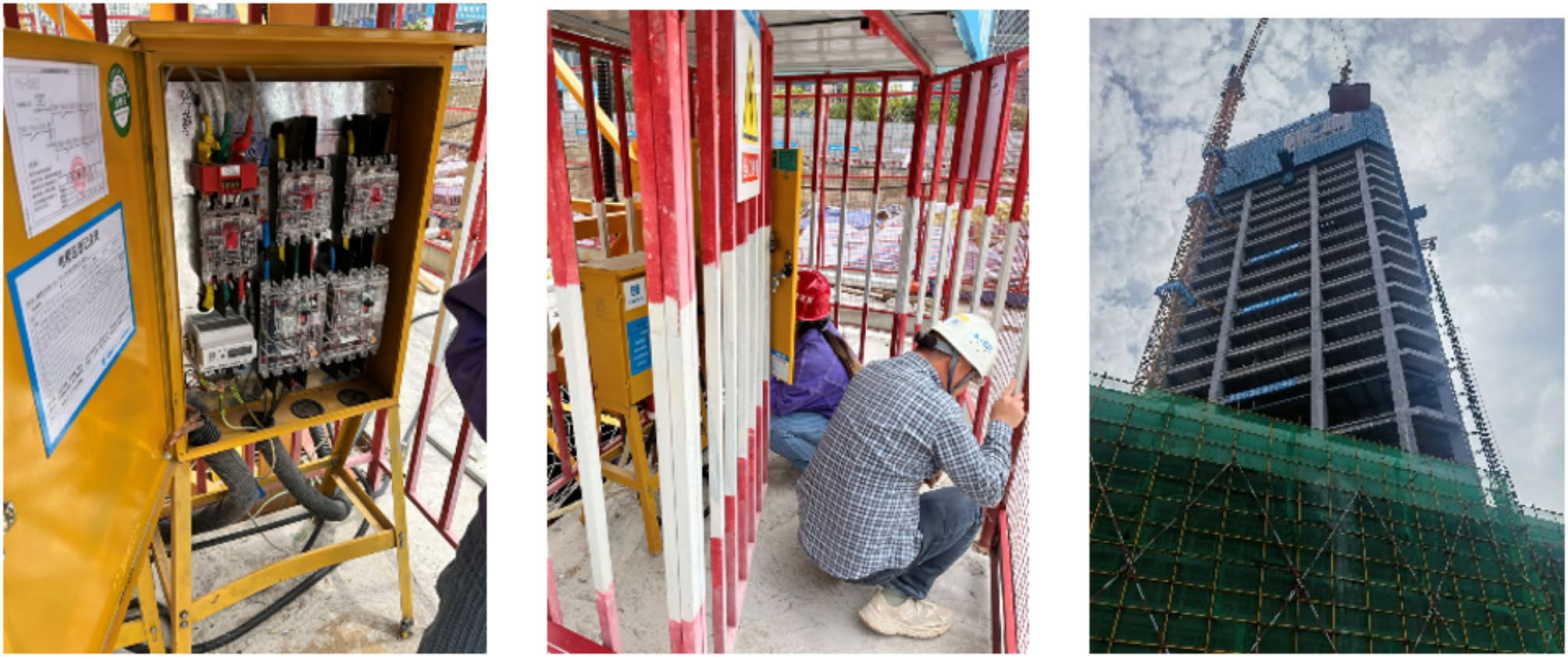
On-site data measurement.

In this study, measured data is used to formulate the calculation model and verify its reliability. The data were sourced from the construction project at Plot B01, Jiangbeizui, Chongqing, China, covering the period from April 27, 2023, to January 31, 2024. The tower crane in this study is of type QTP300, with a torque of 300 kN·m, a maximum working range of 75 m, and a maximum lifting capacity of 2.6t at the end of the arm. Figure 4 provides a brief layout of the construction site and indicates the TC location. TC energy consumption data were measured by the electric meter, with data recording performed by data recorders on-site (Fig. 5).

182 pieces of valid sample were obtained, each containing the duration and the readings of the electricity meter at the start and end of every stage. The mass of building materials corresponding to each sample is in the range of 70 ~ 4000 kg. After data transformation, the values of the seven variables which listed in Table 2 were obtained.

**Table 2:** Dataset of work cycle energy consumption calculation.

Variables	Measurement unit	Description
*t*_LO_	s	The duration of loading stage
*t*_HS_	s	The duration of hoisting stage
*t*_HT_	s	The duration of horizontal transportation stage
*t*_DE_	s	The duration of descending stage
*t*_UL_	s	The duration of unloading stage
*t*_RT_	s	The duration of return stage
*E*	kJ	The energy consumption of a whole work cycle

It is worth mentioning that the measured data exhibits significant heterogeneity, with each variable rejecting the normal distribution hypothesis. Data were measured by the independent measurement method, and arithmetic averaging was employed to eliminate individual recording bias. If the recording bias was longer than 10s, the sample would be discarded. Additionally, considering that extreme values (e.g., *t*_LO_ = 1560s) reflect practical scenarios, removing these data would reduce the model’s generalization ability. Therefore, no subsequent noise reduction treatment was applied. The basic statistics for each variable are shown in Table 3.

**Table 3:** Data of statistical indicators.

Variables (Unit)	Maximum value	Minimum value	Mean value	Standard deviation
*t*_LO_ (s)	1560	4	81.05	127.30
*t*_HS_ (s)	322	1	64.87	56.09
*t*_HT_ (s)	148	10	47.01	26.93
*t*_DE_ (s)	167	5	44.45	27.15
*t*_UL_ (s)	2400	5	121.68	291.14
*t*_RT_ (s)	250	0	90.95	44.94
*E* (kJ)	16941.6	540	4094.02	2797.105

### Partial least squares regression (PLSR)

PLSR was first introduced by Wold et al.^48^. It is a novel multivariate data analysis technique developed for practical applications. PLSR combines key functionalities of Principal Component Analysis (PCA) and Canonical Correlation Analysis (CCA), making it more robust against multicollinearity and noise during regression fitting. Additionally, PLSR is simple, efficient, easy to use, and its computational accuracy is not significantly lower than that of other methods^49,50^.

First, PLSR needs to find the optimal principal component vectors p and q for the independent variable matrix *X* and the dependent variable matrix *Y* respectively. The determination of p and q is inspired by PCA and CCA. PCA provides the projection directions that best capture the differences in the information within the matrix, while CCA maximizes the correlation between the projection vectors of the independent and dependent variable matrices. Based on these requirements, PLSR defines an objective function to solve for the optimal projection vectors:

2 $$\hbox{max} z={[Var\left( {Xp} \right) \times Var\left( {Yq} \right)]^{1/2}}Corr\left( {Xp,Yq} \right)$$

According to the above function, it can be further derived that *p* is the eigenvector corresponding to the largest eigenvalue of *X*^T^*YY*^T^*X* and *q* is the eigenvector corresponding to the largest eigenvalue of *Y*^T^*XX*^T^*Y*.

In fact, since the dependent variable in this study is only the total energy consumption of the TC work cycle, it is evident that *q =* 1.

Second, PLSR needs to solve the coefficient vector *r* for each parameter to satisfy:

3 $$Y=(Xp)r\,+\,K$$

where *K* represents the residual matrix between the predicted values and the true values of the dependent variable. Based on minimizing *||K||*, according to the least squares method, we can solve for:

4 $${\mathbf{r}}=\frac{{{{\mathbf{Y}}^{\text{T}}}({\mathbf{Xp}})}}{{||{\mathbf{Xp}}||}}$$

With *r* known, we can further solve for the residual matrix *K*.

Besides *r*, PLSR also needs to solve for the coefficient vector *c* of the independent variable according to *X* = (*Xp*)*c* + *L*, using the same method to solve for the residual matrix *L*. Using *L* and *K* to replace *X* and *Y* respectively, the next round of solving for *r’*, *L’* and *K’* is performed.

By repeating the above solving process, the result of the PLSR fitting is obtained by summing the coefficient vectors *r* generated from several iterations. Since *n*-dimensional space requires at most *n* linearly independent vectors, the number of iterations mentioned above will not exceed the number of independent variables.

Additionally, considering that including the constant term would be helpful to improve the accuracy of the regression result, Function (1) is adjusted as follows:

5 $$E={\hat {P}_{{\text{LO}}}} \times {t_{{\text{LO}}}}+{\hat {P}_{{\text{HS}}}} \times {t_{{\text{HS}}}}+{\hat {P}_{{\text{HT}}}} \times {t_{{\text{HT}}}}+{\hat {P}_{{\text{DE}}}} \times {t_{{\text{DE}}}}+{\hat {P}_{{\text{UL}}}} \times {t_{{\text{UL}}}}+{\hat {P}_{{\text{RT}}}} \times {t_{{\text{RT}}}}+Con$$

Where *Con* in Function (5) represents the constant term generated during the fitting of the model. It is important to note that, since not calculated from physical relationships, although the unit of power can be used, each $\hat {P}$ in Function (5) should not be interpreted as the average power of each stage without supporting evidence.

## Results

**Fig. 6:**
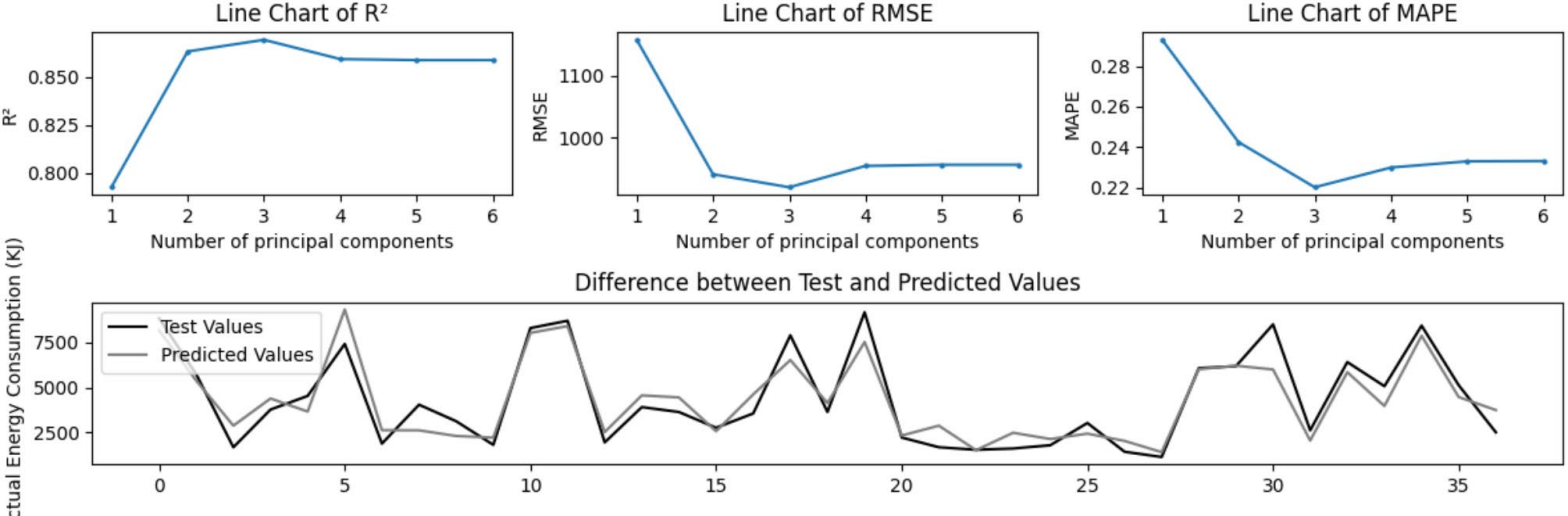
Accuracy of the model under a random training and test set combination.

Among all 182 samples, 146 (80%) were randomly selected for fitting regression coefficients by PLSR method, while the remaining 36 (20%) were used to validate the accuracy of the semi-empirical model. The variation of each accuracy indicator during the fitting process is depicted in Fig. 6.

The energy consumption calculation model corresponding to Fig. 6 is as follows:

6 $$E\,=\,3.38 \times {t_{LO}}+33.59 \times {t_{HS}}+16.11 \times {t_{HT}}+{\text{ }}0.90 \times {t_{DE}}+1.60 \times {t_{UL}}+13.04 \times {t_{RT}} - \,530.63$$

The coefficients of Function (6) indicate that, when the time is extended by the same amount, the impact of descending stage, unloading stage, loading stage, return stage, horizontal transportation stage, and hoisting stage on TC energy consumption increases in this order, with the effect of descending stage potentially being much greater than others.

Subsequently, multiple training and testing sets were randomly generated from the 182 samples, and each training result exhibited high accuracy, with an R² no lower than 0.7 and MAPE no higher than 30%.

## Discussion

Although the test results indicate that Function (5) is a reliable TC energy consumption calculation model, the fitting coefficients require six time-parameters. The complexity of data collection and processing may hinder the model’s application in practical scenarios. To enhance the model’s practicality, this section mainly discusses how to simplify and adjust the modeling parameters.

### Model simplification and accuracy analysis

#### Model simplification

To reduce the variables in Function (5), the regression significance of the six stages were evaluated, using a 95% confidence level as the criterion, and *t*_DE_ was excluded. The *p-value* for each remaining independent variable is shown in Table 4.

**Table 4:** *p-value* for each remaining independent variable.

	*t*_LO_	*t*_HS_	*t*_HT_	*t*_UL_	*t*_RT_
*p*-value	6.92 × 10^− 9^	5.98 × 10^− 42^	3.12 × 10^− 4^	2.36 × 10^− 6^	9.78 × 10^− 10^

**Fig. 7:**
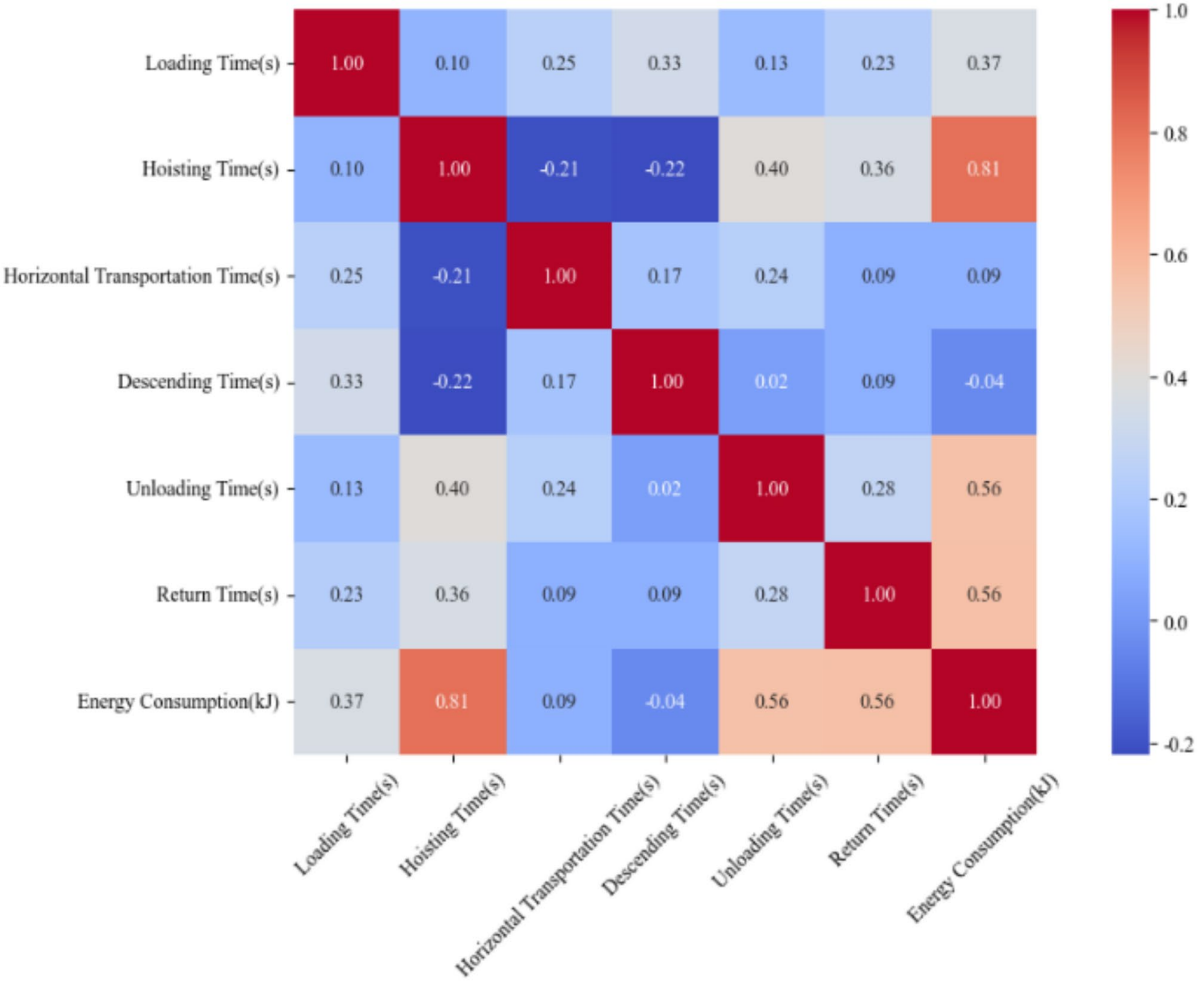
Correlation heatmap of each independent variable (stage) and dependent variable (energy consumption).

**Fig. 8:**
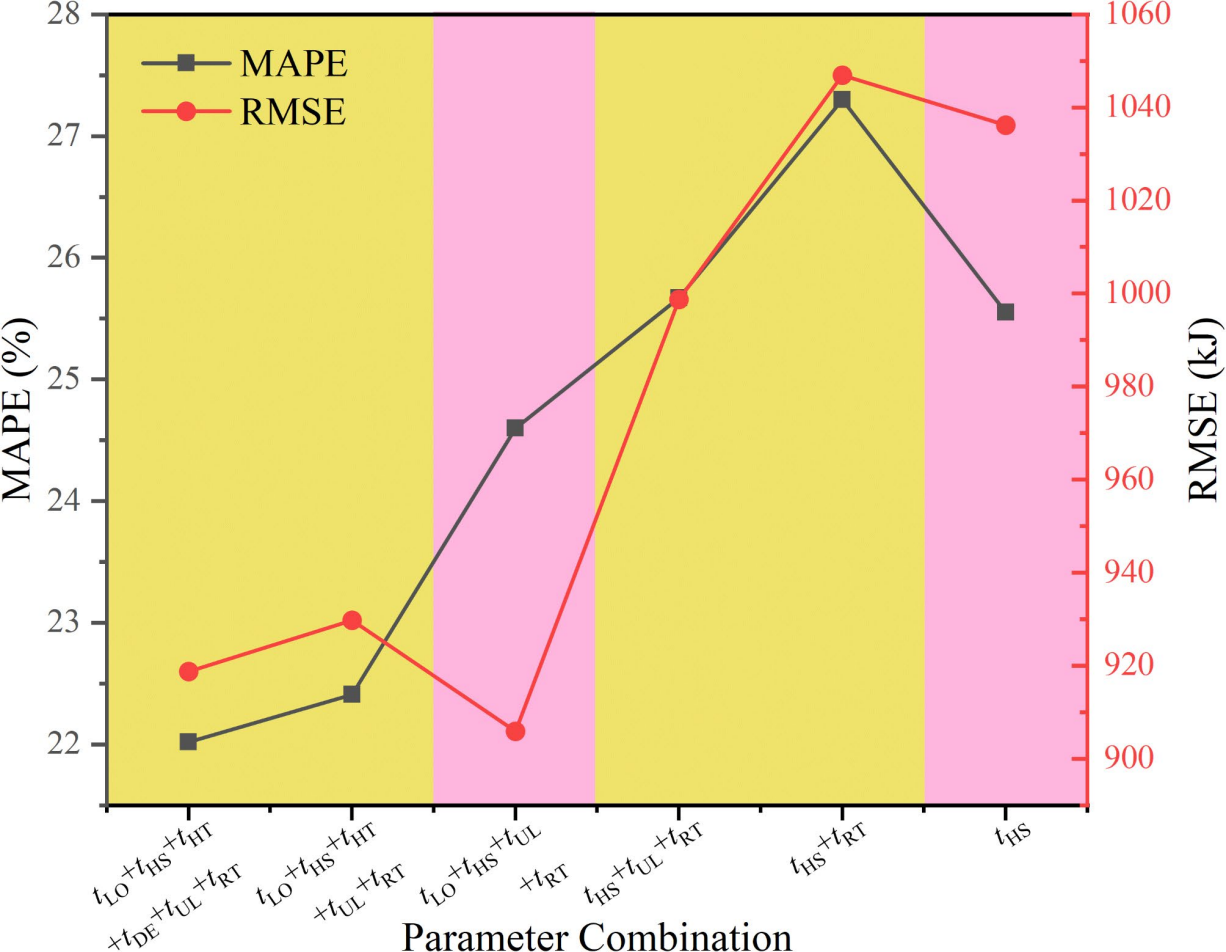
Error variation in the model simplification process.

The independent variables, which is recorded in Table 4, were removed based on their correlation with TC energy consumption from smallest to largest, as shown in Fig. 7. The remaining variables were then fitted using PLSR method. The energy consumption calculation accuracy of each version is detailed in Fig. 8; Table 5. The utilized training set was similar to the set of Function (6).

**Table 5:** Calculation models’ accuracy for each variable combination.

Independent variables	RMSE	MAPE (%)
*t*_LO_ + *t*_HS_ + *t*_HT_ + *t*_DE_ + *t*_UL_ + *t*_RT_	918.79	22.02
*t*_LO_ + *t*_HS_ + *t*_HT_ + *t*_UL_ + *t*_RT_	929.78	22.41
*t*_LO_ + *t*_HS_ + *t*_UL_ + *t*_RT_	905.93	24.60
*t*_HS_ + *t*_UL_ + *t*_RT_	998.70	25.67
*t*_HS_ + *t*_RT_	1046.90	27.30
*t*_HS_	1036.19	25.55

The results in Table 5 indicate that using either *t*_LO_, *t*_HS_, *t*_UL_ and, *t*_RT_ or only *t*_HS_ for constructing the semi-empirical consumption model can keep a high level of accuracy in the calculations. The former (referred to as Most-Accurate Model hereafter) offers higher precision, while the latter (referred to as Most-Simplified Model hereafter) requires the least variables.

#### Accuracy comparison of models

In practice or research, three models are commonly used for calculating TC energy consumption:

- Empirical Model: This model is expressed as “Mechanical Rated Power × Mechanical Operation Time = Energy Consumption”. The Mechanical Rated Power can be found in industry quotas, and this model is mainly used during the design phase of a project to estimate the general TC energy consumption.
- Mechanism-based Model: This model is expressed as “Σ(Sub-Mechanism Power × Sub-Mechanism Operating Time) = Energy Consumption”. Manufacturers’ manuals typically provide the power data for sub-mechanisms. This model is less commonly used, but several studies on TC mechanisms have employed it^51,52^.
- Whether-Loaded Model: This model divides TC operation process into loaded and unloaded states, which expressed as “Loaded Power × Loaded time + Unloaded power ×Unloaded time = Energy Consumption”. This model has been used in some recent TC studies^15,16^. The power for both states is estimated^16^, and no literature has provided specific values for these estimates.

**Fig. 9:**
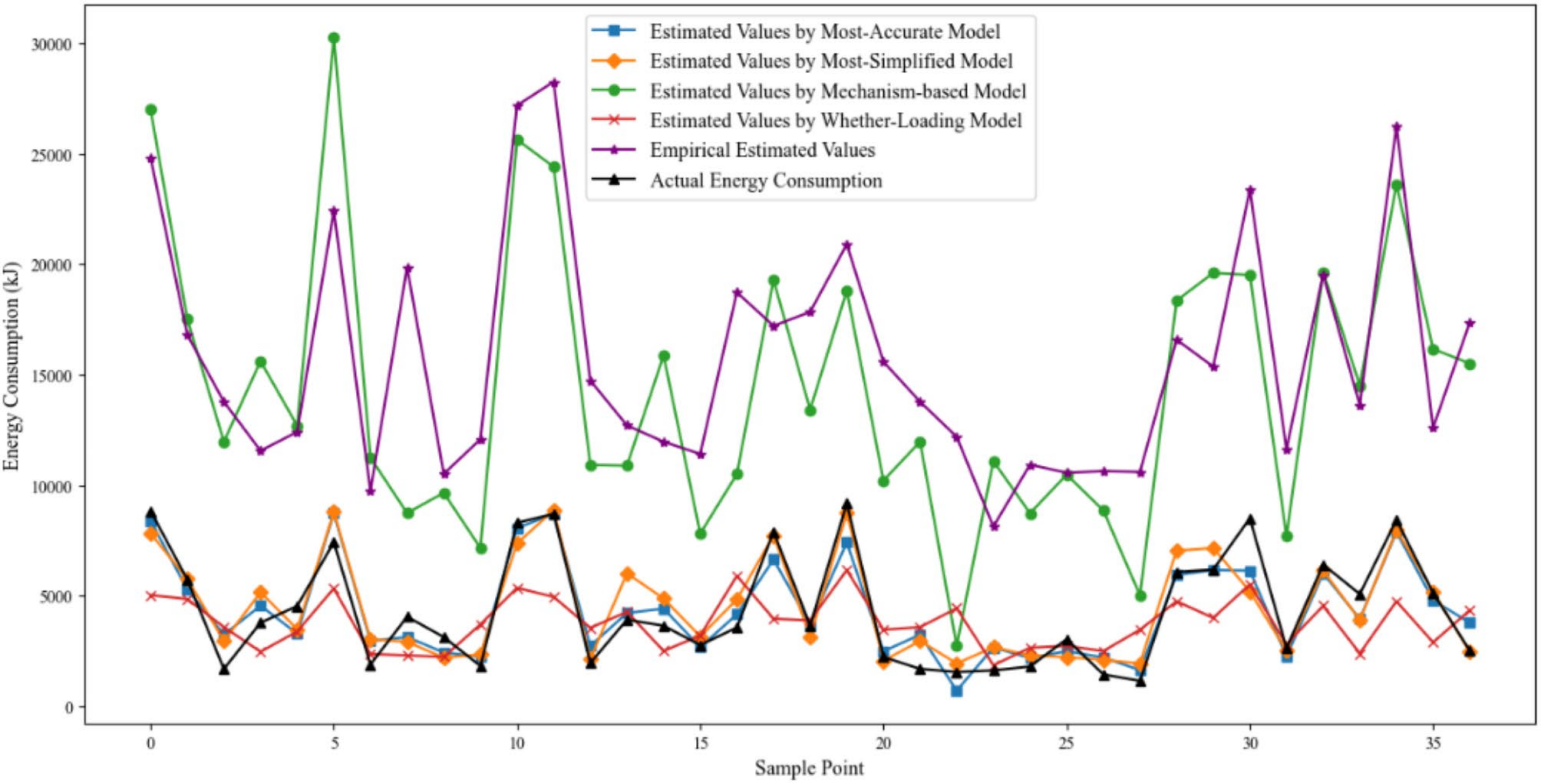
Energy consumption forecast results of different methods.

The power coefficients for the three models are shown in Table 6. Figure 9; Table 7 illustrate the accuracy indicators of the above models and the proposed models.

**Table 6:** Coefficients of different calculation models for QTP300.

Models	Coefficients	Values (kW)	References
Empirical model	Total power	36.95	*Chongqing Construction Engineering Machinery Quota* (*CQJXDE-2018*)
Mechanism-based model	Hoisting mechanism power	75	The instruction manual of QTP300
	Slewing mechanism power	9	
	Trolleying mechanism power	7.5	
Whether-loaded model	Loaded power	9.52	Regression fitted by the training set
	Unloaded power	4.97	

**Table 7:** Model prediction error.

Models	MAPE (%)	RMSE (kJ)
Most-accurate model	24.60	905.93
Most-simplified model	25.55	1036.19
Whether-loaded model	39.78	2136.29
Mechanism-based Model	279.64	10906.54
Empirical model	351.07	11919.10

Figure 9 and Table 7 show that traditional models (Empirical Model and Mechanism-based Model) perform poorly in practical scenarios and can only be used for very rough energy consumption estimates. In contrast, the simplified model proposed in this study achieves a significant improvement in calculation accuracy.

The failure of the Empirical Model in terms of accuracy suggests that summarizing the power variation during TC operation process with a fixed power value is unreasonable. The accuracy remained so poor, even when reconstructed TC’s total power using the training set, and it confirms the above viewpoint.

It must be acknowledged that the time data recorded in this study came from observations of TC external motion state, which were likely to negatively impact the fitting and calculation of the Mechanism-based Model. Therefore, it would be unjustified to assert that the Mechanism-based Model is unsuitable for rigorous energy consumption studies and analyses. However, from the practical application ability, the simplified model proposed in this study has a significant advantage.

Although the Whether-Loaded Model demonstrated good computational accuracy, its R² value was only 0.29, indicating poor model fit. Considering that the lack of a constant term might have contributed to the low fit, we added a constant term and rebuilt the model. However, the R² value and estimation ability of the model did not improve significantly. Since the Most-Accurate Model and Most-Simplified Model constructed with the same training set both yielded R² values close to 1 (0.87 and 0.83, respectively), it is believed that the poor fit of the Whether-Loaded Model is not due to data noise or insufficient sample size. This suggests that whether under the loaded state is not a key factor influencing TC energy consumption.

#### Analysis of most-simplified model

The Most-Simplified Model is both simple and accurate enough. It can be expressed as:

7 $$E={\hat {P}_{{\text{HS}}}}^{\prime } \times {t_{{\text{HS}}}}+{E_{{\text{extra}}}}$$

In Function (7), ${\hat {P}_{{\text{HS}}}}^{\prime }$ (kW) and *E*_extra_​ (kJ) represent the empirical power and constant term of the Most-Simplified Model obtained through PLSR method, respectively.

**Fig. 10:**
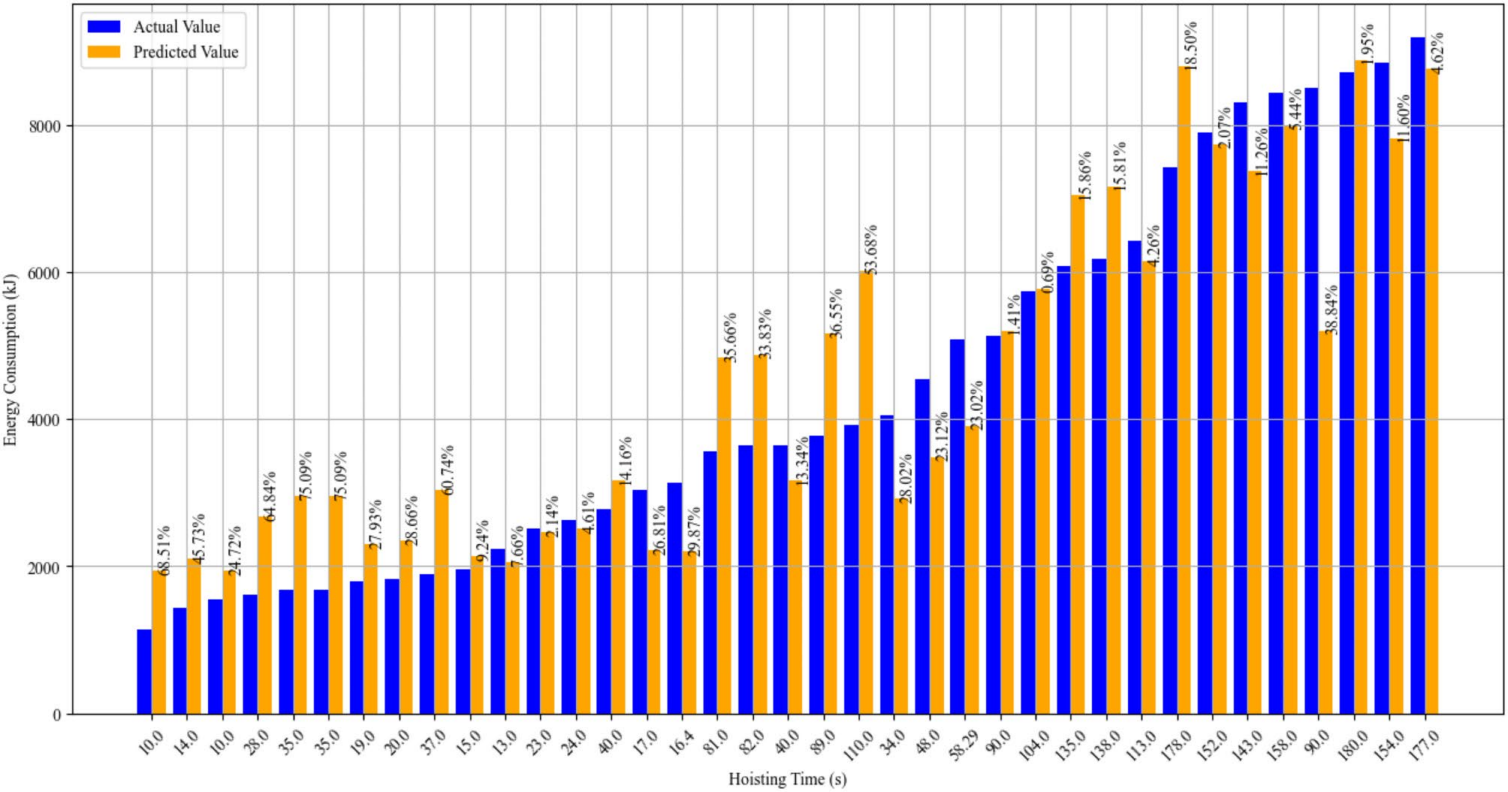
Energy consumption prediction errors of Function (7).

Figure 10 shows the error of the Most-Simplified Model’s energy consumption prediction results for the test set.

*The bars are arranged from left to right according to the actual energy consumption, from smallest to largest.

* The percentages above the prediction bars indicate the relative error Δ, i.e., the difference between the actual and predicted values. $\Delta =\frac{{|Predict - Actual|}}{{Actual}} \times 100\%$

Although Function (7) demonstrates considerable accuracy in calculating the tower crane’s energy consumption, Fig. 10 indicates that when the *t*_HS_ is short or the work cycle’s energy consumption is low, the prediction error of Function (7) may be relatively large.

### Analysis of the relationship energy consumption and other factors

In this section, Function (7) is transformed based on the physical relationships of TC hoisting stage to eliminate parameters such as *t*_HS_, which need to be measured during TC work cycle, and analyze the key engineering parameters affecting TC energy consumption at the construction site.

#### Transformation of energy consumption function

**Fig. 11:**
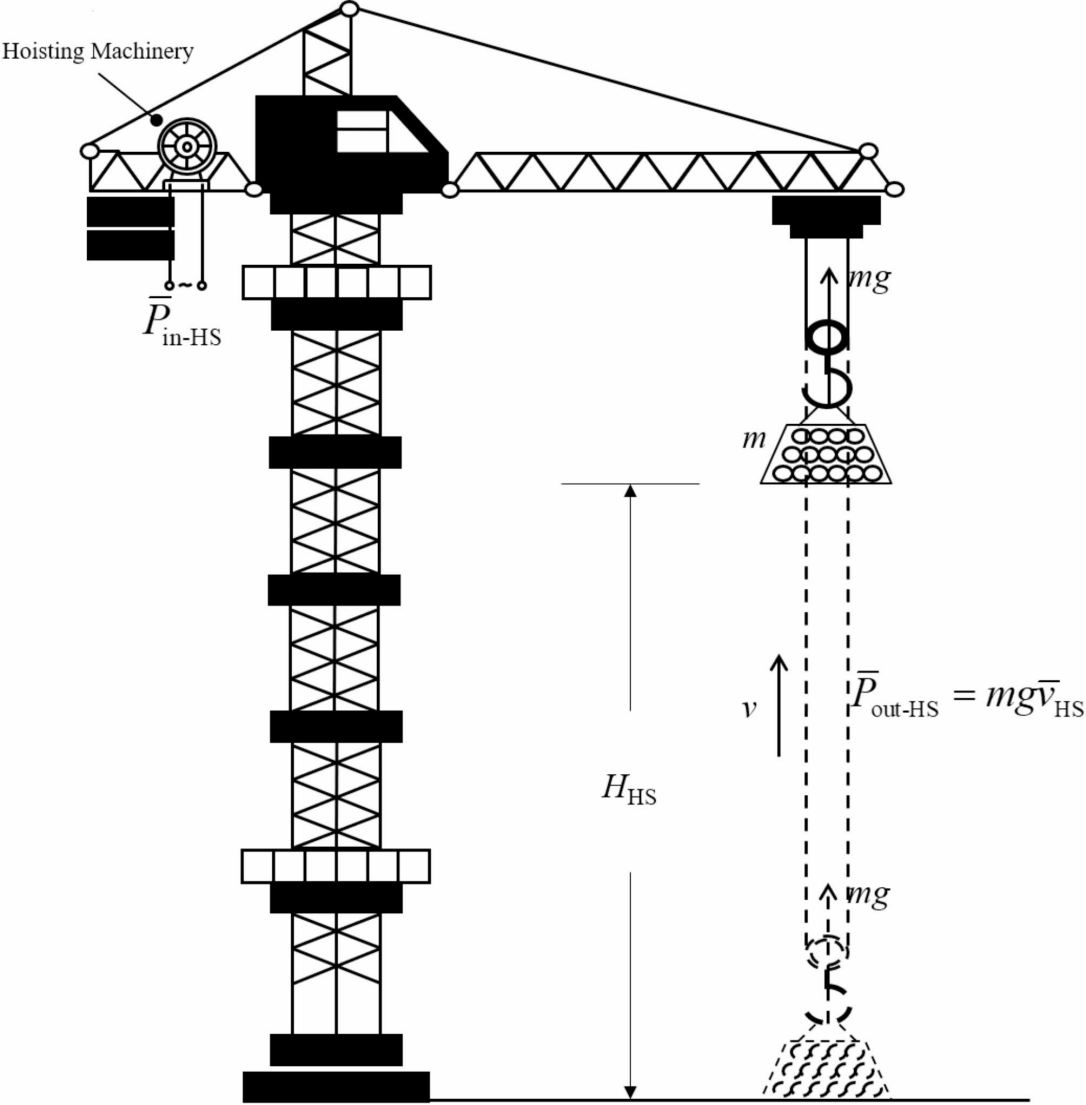
Diagram of TC hoisting stage.

Figure 11 is a visual representation of the hoisting stage in TC work cycle. To make the transformation of the energy consumption function more understandable, Fig. 11 records some of the parameters that will appear in the following equations.

Let the total hoisting height of *t*_HS_ be *H*_HS_ (m), and the average hoisting speed be ${\bar {v}_{{\text{HS}}}}$ (m/s). Replacing *t*_HS_ with ${H_{{\text{HS}}}}/{\bar {v}_{{\text{HS}}}}$ in Function (7) gives:

8 $$E={\hat {P}_{{\text{HS}}}}^{\prime } \times \frac{{{H_{{\text{HS}}}}}}{{{{\bar {v}}_{{\text{HS}}}}}}+{E_{{\text{extra}}}}$$

Then eliminating ${\bar {v}_{{\text{HS}}}}$ became the next key task.

The average output power during the TC hoisting stage (${\overline {P} _{{\text{out-HS}}}}$, unit: kJ) is given by the following function:

9 $${\overline {P} _{{\text{out-HS}}}}=Fv \Rightarrow \frac{1}{{1000}} \times mg{\bar {v}_{{\text{HS}}}}$$

In Function (9), *m* (kg) is the mass of the loaded building material, and *mg* represents its weight, corresponding to *F*.

Based on the relationship between the input power (${\overline {P} _{{\text{in-HS}}}}$, unit: kJ) of the hoisting mechanism and ${\overline {P} _{{\text{out-HS}}}}$, we get:

10 $${\overline {P} _{{\text{out-HS}}}}={\eta _{{\text{HS}}}}{\overline {P} _{{\text{in-HS}}}}={\eta _{{\text{HS}}}} \times \frac{{{E_{{\text{HS}}}}}}{{{t_{{\text{HS}}}}}}$$

In Function (10), *η*_HS_ (%) represents the proportion of the hoisting mechanism’s output power during the hoisting stage relative to the total energy consumption. *E*_HS_ (kJ) is the energy consumption during the hoisting stage.

Additionally, it was found that *E*_HS_ can be calculated using the following semi-empirical model (see the Appendix for details):

11 $${E_{{\text{HS}}}}={\hat {P}_{{\text{HS}}}}^{{\prime \prime }} \times {t_{{\text{HS}}}} - {E_{{\text{extra-HS}}}}$$

Similar with Function (7), ${\hat {P}_{{\text{HS}}}}^{{\prime \prime }}$ (kW) and *E*_extra−HS_ (kJ) are both empirical coefficients.

By combining Function (9), (10), and (11), and replacing *t*_HS_ with ${H_{{\text{HS}}}}/{\bar {v}_{{\text{HS}}}}$, the following function is obtained:

12 $${\bar {v}_{{\text{HS}}}}=\frac{{{\eta _{{\text{HS}}}}{{\hat {P}}_{{\text{HS}}}}^{{\prime \prime }}}}{{\frac{1}{{1000}} \times mg+{\eta _{{\text{HS}}}}\frac{{{E_{{\text{extra-HS}}}}}}{{{H_{{\text{HS}}}}}}}}$$

Substituting Function (12) into Function (8), the energy consumption (*E*) calculation function for TC work cycle can be written as:

13 $$E=\frac{1}{{1000}} \times \frac{{{{\hat {P}}_{{\text{HS}}}}^{\prime }}}{{{\eta _{{\text{HS}}}}{{\hat {P}}_{{\text{HS}}}}^{{\prime \prime }}}} \times mg{H_{{\text{HS}}}}+\frac{{{{\hat {P}}_{{\text{HS}}}}^{\prime }}}{{{{\hat {P}}_{{\text{HS}}}}^{{\prime \prime }}}}{E_{{\text{extra-HS}}}}+{E_{{\text{extra}}}}$$

#### Analysis of the energy consumption calculation function

According to Function (13), when the TC model (${\hat {P}_{{\text{HS}}}}^{\prime }$, ${\hat {P}_{{\text{HS}}}}^{{\prime \prime }}$, *η*_HS_, *E*_extra_, and *E*_extra−HS_) and the hoisting height (*H*_HS_) are determined, the energy consumption of the TC work cycle (*E*) has a linear relationship with the mass (*m*) of the lifted item, and *E* increases as *m* increases.

Assuming the total mass of items at the material yard is *M* (kg), the mass of each building material is *m*_i_​ (kg), and the tower crane completes all tasks in *N* work cycles, the total energy consumption for all work cycles (*E*_total_, unit: kJ) is:

14 $$\begin{gathered} {E_{{\text{total}}}}=\sum\limits_{{i=1}}^{N} {{E_{\text{i}}}} =\sum\limits_{{i=1}}^{N} {(\frac{1}{{1000}} \times \frac{{{{\hat {P}}_{{\text{HS}}}}^{\prime }}}{{{\eta _{{\text{HS}}}}{{\hat {P}}_{{\text{HS}}}}^{{\prime \prime }}}} \times {m_{\text{i}}}g{H_{{\text{HS}}}}+\frac{{{{\hat {P}}_{{\text{HS}}}}^{\prime }}}{{{{\hat {P}}_{{\text{HS}}}}^{{\prime \prime }}}}{E_{{\text{extra-HS}}}}+{E_{{\text{extra}}}})} \\ =\frac{1}{{1000}} \times \frac{{{{\hat {P}}_{{\text{HS}}}}^{\prime }}}{{{\eta _{{\text{HS}}}}{{\hat {P}}_{{\text{HS}}}}^{{\prime \prime }}}} \times (\sum\limits_{{i=1}}^{N} {{m_{\text{i}}}} ) \times g{H_{{\text{HS}}}}+N(\frac{{{{\hat {P}}_{{\text{HS}}}}^{\prime }}}{{{{\hat {P}}_{{\text{HS}}}}^{{\prime \prime }}}}{E_{{\text{extra-HS}}}}+{E_{{\text{extra}}}}) \\ =\frac{1}{{1000}} \times \frac{{{{\hat {P}}_{{\text{HS}}}}^{\prime }}}{{{\eta _{{\text{HS}}}}{{\hat {P}}_{{\text{HS}}}}^{{\prime \prime }}}} \times Mg{H_{{\text{HS}}}}+N(\frac{{{{\hat {P}}_{{\text{HS}}}}^{\prime }}}{{{{\hat {P}}_{{\text{HS}}}}^{{\prime \prime }}}}{E_{{\text{extra-HS}}}}+{E_{{\text{extra}}}}) \\ \end{gathered}$$

In Function (14), *E*_i_ represents the energy consumption for each work cycle. Function (14) shows that, when all other parameters are fixed, *E*_total_ is a monotonically increasing linear function of *N*. This means that to reduce the total energy consumption of the tower crane, the crane should complete the transportation tasks in as few work cycles as possible. Additionally, *E*_total_ is also a monotonically increasing linear function of *M*, which indicates that when other parameters are fixed, the larger the total mass of items to be lifted at a supply point, the less energy consumption for completing all lifting tasks.

The application of Function (14) in construction management can be illustrated using a simple case.

The construction of high-rise concrete structures can be summarized into the following three modes:

##### Full cast-in-place mode

This mode requires TC to transport items such as formwork, reinforcement, and concrete (with a bucket) in phases according to the construction process and schedule. The total load mass is *M*_Cast−in_, and the total number of work cycles is *N*_Cast−in_.

##### Precast construction mode

This mode uses prefabricated components (PC) produced in a factory and directly hoisted at the construction site. Due to the significant reduction in the need for formwork and supports, the total mass of items lifted by the crane in this mode is *M*_PC_ < *M*_Cast−in_. Additionally, because the reinforcement and concrete are integrated into a single PC element, the number of work cycles required by the crane is *N*_PC_ < *N*_Cast−in_.

##### Modular construction (MiC) mode

The essence of MiC is assembling room units produced in a factory to form a complete building. In this mode, several PCs are combined into a single unit. Some of the construction processes, such as component connection, are omitted. In this mode, the number of work cycles is *N*_MiC_ < *N*_PC_, and the total mass of items is *M*_MiC_ = *M*_PC_.

Once the TC model, location, and material storage yard position are determined, and assuming there is only one material yard, while ensuring the crane’s load capacity meets the requirements for all items, Function (14) can be used to obtain the following relationship:

*E*_Cast-in_ > *E*_PC_ > *E*_MiC_ (15).

Where *E*_Cast−in_, *E*_PC_, and *E*_MiC_ represent the total energy consumption for TC of the same type to complete all the lifting tasks for one room unit under Full Cast-in-Place Mode, Precast Construction Mode, and MiC Mode, respectively. Inequality (15) indicates that MiC Mode is the most effective in reducing energy consumption on the construction site.

## Theoretical and practical implications

This study fills several gaps in the existing research. It designs a semi-empirical energy consumption calculation model for TC. As a significant improvement in computational accuracy, the new model provides a more reliable foundation for TC energy consumption calculations. At the same time, this study highlights the drawbacks of traditional research^15,16,23^, which has used total operation time or loaded and unloaded time as decision variables, and points out that hoisting time, load mass, hoisting height, and the number of work cycles are more crucial parameters that deserve attention in TC energy consumption research.

Valuable references are offered for future exploration. The study confirms the feasibility of constructing energy consumption models by dividing construction machinery operational stages and using PLSR methods to fit model coefficients in construction machinery energy consumption studies. Additionally, it proposes the energy consumption-time-other engineering parameter relationship chain to extract key parameters for energy consumption. The chain could be applied to energy consumption research for other types of construction machinery.

**Fig. 12:**
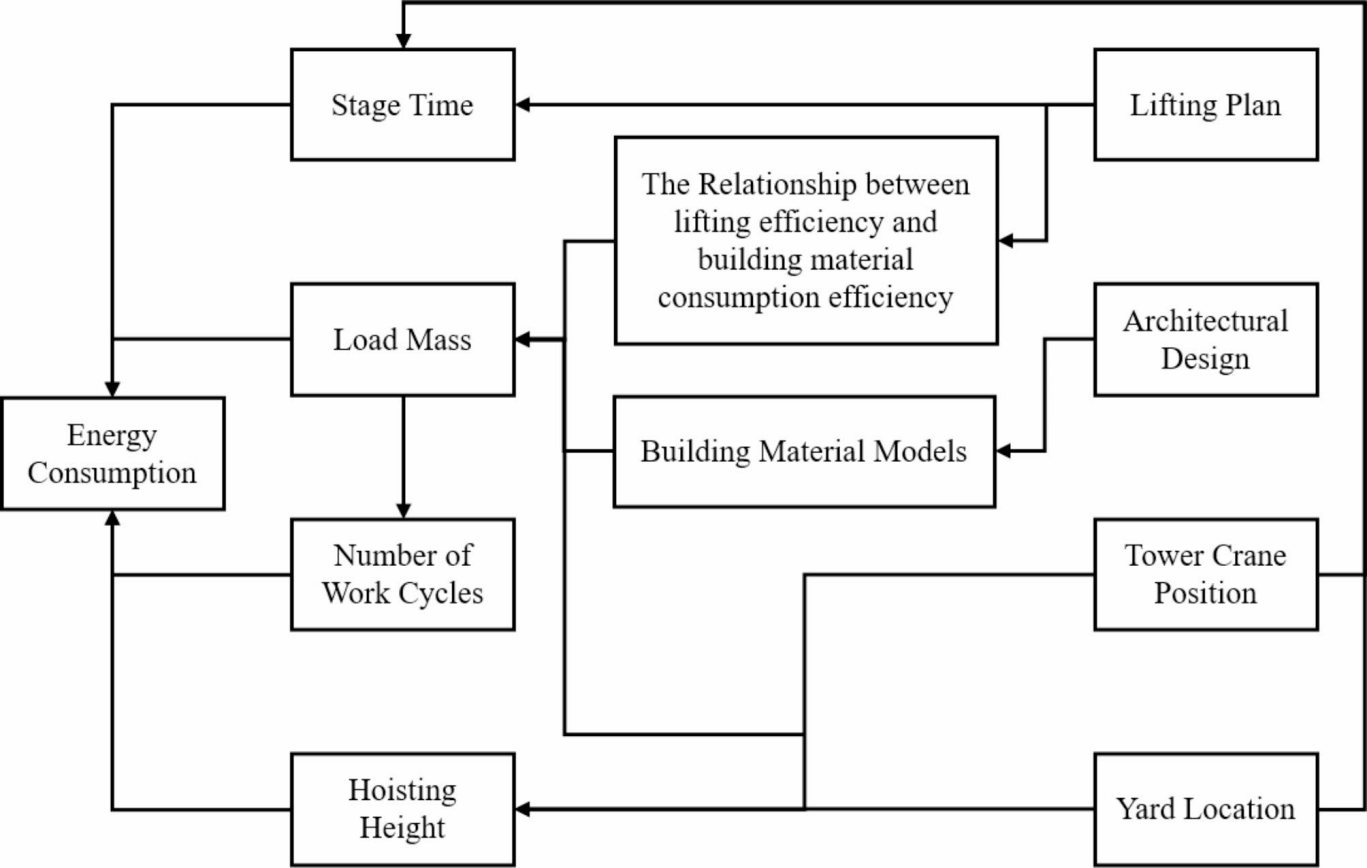
Relationship between research subjects.

The practical implications of this study are manifold. The computational parameters for the energy consumption model can be directly observed and measured, which eliminates the need for inference and analysis of TC internal mechanisms in complex construction scenarios. Combined with machine vision technology, data collection can be carried out more efficiently. Moreover, the amount of basic data required for model construction is not large, meaning that construction companies can quickly obtain quota data for various TC models, thereby improving their energy consumption and cost management capability. Furthermore, based on the key factors extracted in this study, a parameter relationship network, as shown in Fig. 12, can be constructed, helping managers to more comprehensively consider questions such as “Where should the tower crane be placed?“, “Where should the material storage yard be located?“, and “Which order should the materials be hoisted?”^13,53–55^ Finally, from the perspective of reducing construction machinery energy consumption, this study highlights the advantages of buildings with high prefabrication rates. This provides a new basis for promoting modular and prefabricated construction.

## Conclusions

In this study, a new semi-empirical energy consumption calculation model for TC was proposed. TC operation process was divided into a number of work cycles, each consisting of six consecutive stages. By using measured time data and energy consumption data for each stage, the model was constructed and its accuracy was validated. The successful construction of the energy consumption calculation model based on operational stage division provides suggestions for improving the accuracy of energy consumption models for other types of construction machinery.

One of the key innovations in this study is that the field-measured data enhanced the model’s ability to reflect real-world patterns, thereby improving its generalization capability. However, the small data size also limited the ability to support advanced machine learning algorithms for model training. The use of the PLSR method overcame the challenge of small sample sizes, allowing for the construction of a highly accurate energy consumption calculation model. In the field where data collection is difficult, especially for large construction machinery, this successful advancement highlights the potential application of statistical regression methods like PLSR in small sample model developing. Additionally, PLSR’s excellent noise resistance and high computational efficiency further demonstrate its suitability for construction machinery research.

Regarding energy consumption calculation, four parameters were emphasized: hoisting time, load mass, number of work cycles, and hoisting height. These parameters identified the key objects for TC energy consumption control. At the same time, serving as critical points in TC scheduling and design research, they link energy consumption to these topics, contributing to the comprehensiveness of multi-objective optimization problems in TC management. In this study, other key engineering parameters (material quality, number of hoisting cycles, and hoisting height) were derived from the physical transformation of the stage time in semi-empirical model, offering a stable approach to extracting key engineering parameters for other construction machinery.

The proposed model improves the simplicity and accuracy of TC energy consumption calculation, providing a computational and modeling foundation for future research. Meanwhile, the discussion and identification of key parameters provide insights for advancing TC research.

Despite several breakthroughs, this study has limitations. The first limitation is the data size. Due to the small sample size, the PLSR method was used, which restricted the exploration of nonlinear relationships between variables. About another limitation, the study revealed that energy consumption is significantly associated with the TC specification, but the single research subject in this study doesn’t allow for an analysis of the specific impact process. Additionally, for TC with larger slewing and trolleying mechanism power, it is unclear whether the simplified model would change.

To overcome these limitations, the core task is to increase the sample size and variety. Therefore, future research should focus on the development of automatic engineering data collection technologies (e.g., machine vision and wireless communication technologies). With sufficient samples, it would be possible to explore the relationship between different specifications and TC energy consumption. Furthermore, more advanced machine learning methods, such as Random Forest and SVR, could be used to analyze potential nonlinear relationships, thereby achieving a comprehensive improvement in the research of TC energy consumption.

## Electronic supplementary material

Below is the link to the electronic supplementary material.

Supplementary Material 1

## Data Availability

The datasets analysed during the current study are not publicly available due to the involvement of construction company machinery usage costs but are available from the corresponding author on reasonable request.
