# Supplementary material for: A parameterized model for tower crane energy consumption was developed based on theoretical formulation and field data
Source: Sci Rep. 2025 Mar 26;15:10453. doi: 10.1038/s41598-025-94875-5 (PMC11947251; doi:10.1038/s41598-025-94875-5)
Supplement: Supplementary file 1 — Supplementary Material 1 [file 41598_2025_94875_MOESM1_ESM.docx]

**Appendix**

Using the raw data from 182 samples, the energy consumption of hoisting stage (*E*_HS_) for each sample was calculated. **Table A1** shows the statistical indicators.

**Table A1** Statistical indicators of *t*_HS_ and *E*_HS_

| Variables (Unit) | Maximum Value | Minimum Value | Mean Value | Standard Deviation |
| --- | --- | --- | --- | --- |
| *t*_HS_ (s) | 322 | 1 | 64.87 | 56.09 |
| *E*_HS_ (kJ) | 13568.4 | 0 | 2152.48 | 2324.45 |

The following function was fitted using statistical regression:

$E_{\text{HS}}=\hat{P}_{\text{HS}}^{' '}\times t_{\text{HS}}-E_{\text{extra-HS}}$ (11)

**Fig. A1** shows the accuracy of **Function (11)**.


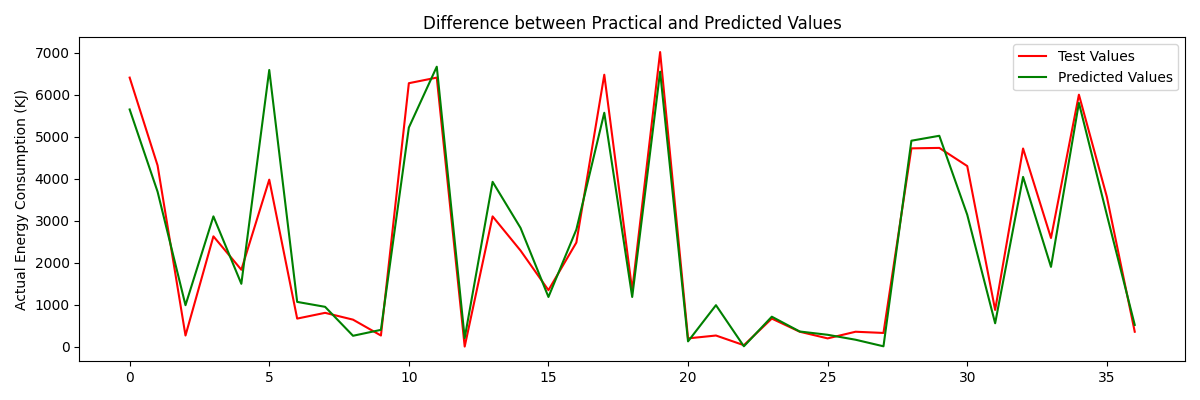


**Fig. A1.** Accuracy of **Function (11)** (RMSE = 653.03 kJ, R² = 0.92)

The hoisting stage of several work cycles is short, and the readings of the stage's start and end are consistent, resulting in *E*_HS_ = 0 in some samples, which made it impossible to calculate MAPE.

In the multiple fitting results, R² is no less than 0.84.

Additionally, since the constant term in all fitting results is negative (**Fig. A2**), to ensure that *E*_extra-HS_ is positive, the constant term in Function (11) is written as -*E*_extra-HS_.


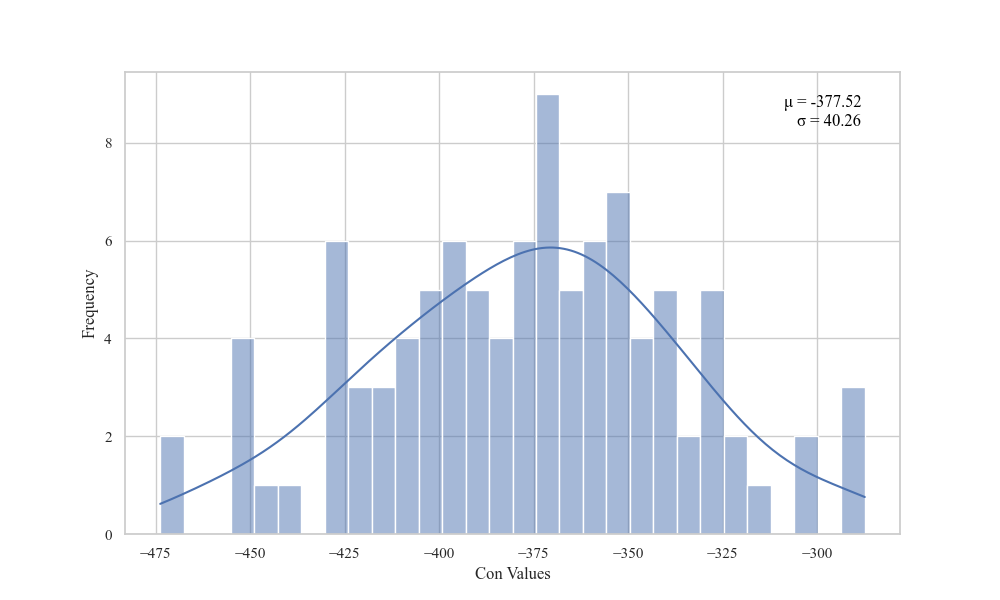


**Fig. A2.** Distribution of constant terms in the multiple fitting results
